# A Gut Feeling: The Importance of the Intestinal Microbiota in Psychiatric Disorders

**DOI:** 10.3389/fimmu.2020.510113

**Published:** 2020-10-19

**Authors:** Javier Ochoa-Repáraz, Christina C. Ramelow, Lloyd H. Kasper

**Affiliations:** ^1^ Department of Biology, Eastern Washington University, Cheney, WA, United States; ^2^ Department of Microbiology and Immunology, Geisel School of Medicine at Dartmouth College, Hanover, NH, United States

**Keywords:** microbiome, neurological disorders, gut/brain axis, neuroinflammation, immunoregulation

## Abstract

The intestinal microbiota constitutes a complex ecosystem in constant reciprocal interactions with the immune, neuroendocrine, and neural systems of the host. Recent molecular technological advances allow for the exploration of this living organ and better facilitates our understanding of the biological importance of intestinal microbes in health and disease. Clinical and experimental studies demonstrate that intestinal microbes may be intimately involved in the progression of diseases of the central nervous system (CNS), including those of affective and psychiatric nature. Gut microbes regulate neuroinflammatory processes, play a role in balancing the concentrations of neurotransmitters and could provide beneficial effects against neurodegeneration. In this review, we explore some of these reciprocal interactions between gut microbes and the CNS during experimental disease and suggest that therapeutic approaches impacting the gut-brain axis may represent the next avenue for the treatment of psychiatric disorders.

## Introduction

Charles Darwin kept a diary where he would annotate feelings and symptoms, often describing his trouble with the gastrointestinal (GI) tract and anxiety (1). In one of his letters to his medical advisors, he noted the “nervousness” when his wife Emma would depart that would trigger “intensely acid, slimy (sometimes bitter) vomit”. He also wrote in The Expression of the Emotions in Man and Animals (1872): “The manner in which the secretions of the alimentary canal and certain other organs … are affected by strong emotions, is another excellent instance of the direct action of the sensorium on these organs, independently of the will or of any serviceable associated habit” (2), as was recently discussed in the context of the gut/brain axis (3), the topic of this review article.

Is there a scientific basis for the adage, “my gut tells me?” Reading Darwin’s notes, one would consider that emotions and GI tract functions are directly connected. As the most recent works demonstrate, the intestinal tract is home for a heterogeneous microbial ecosystem dominated by bacteria but also comprised of viruses, archaea, and other eukaryotic microorganisms. Although the existence of this complex intestinal ecosystem was first alluded to by the Nobel laureate Eli Mechnikov over a century ago, the biological relevance of the large variety of microbial species that colonize our intestines soon after birth has been understudied and certainly unappreciated. With increasing attention worldwide, the potential impact that the gut microbiome has on human health and disease has now begun to be understood.

**Figure 1:**
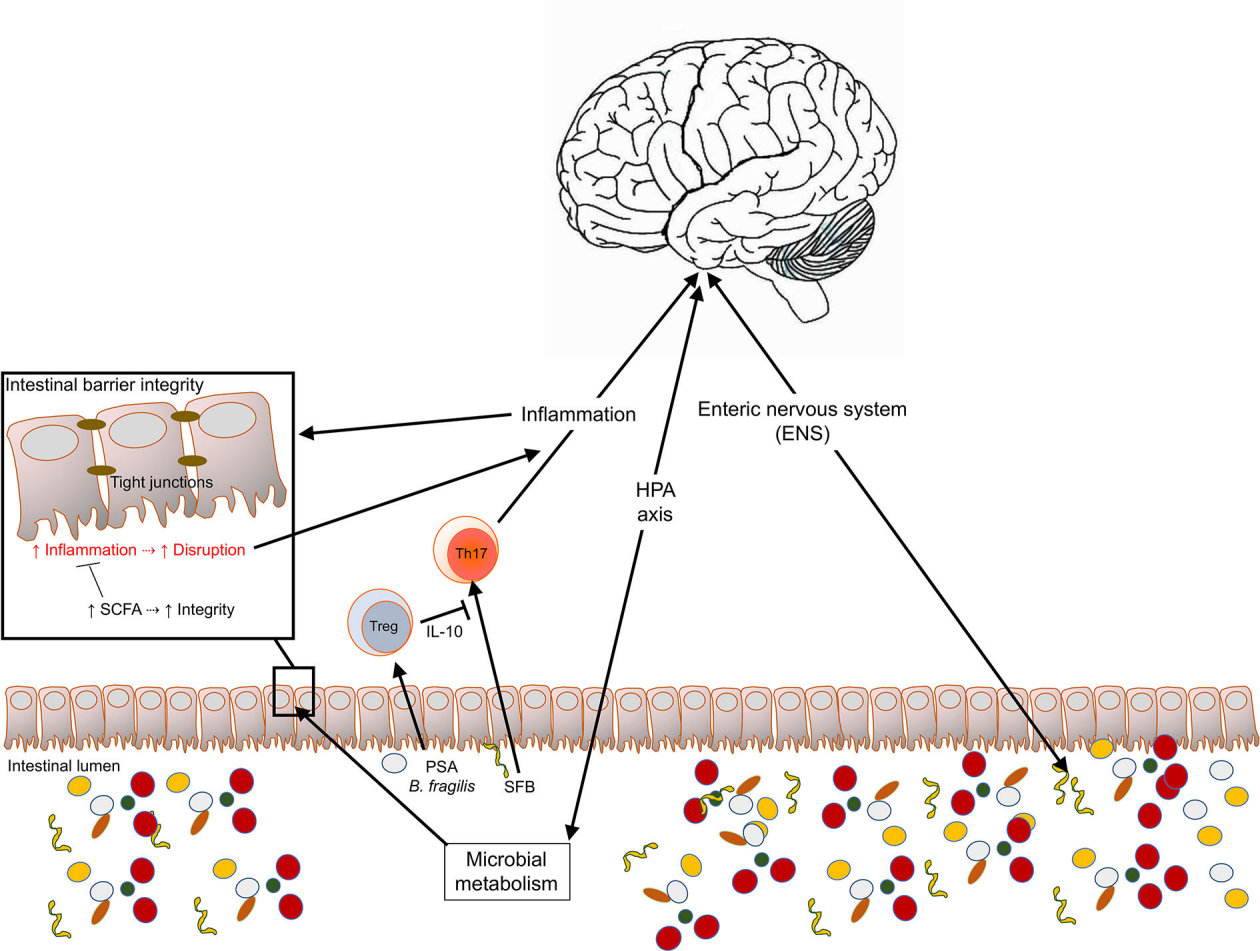
Multifactorial interactions occurring between the intestinal microbiota and products and the CNS. The CNS and the gut microbiota are connected reciprocally through the hypothalamic-pituitary-adrenal (HPA) axis, the immune system, and the neuronal system. These interactions have critical impacts on immune homeostasis, intestinal barrier permeability and the integrity of the blood brain barrier and can regulate CNS inflammation and disease.

Multiple factors affect the composition of the gut microbiome, diet being possibly the most prominent (4). Other environmental exposures such as pollution, antibiotic over-usage, lifestyle habits, vitamin D skin exposure, and a range of other factors such as host genetic composition have been shown to promote changes in the intestinal microbiome (5). Because of microbiome and host’s multifactorial interactions, these changes can lead to significant functional changes on the immune, metabolic or neuroendocrine systems, that could potentially result in disease. Dysbiosis, the mechanism of disease triggered by the microbial imbalance of normal gut microbial colonization, may be the source for a wide range of human conditions (6). Increasing number of reports now show that alterations of the gut microbiome, in some cases observed at the lowest taxonomic levels, are detected in stool samples obtained from patients suffering from metabolic (7–9) and autoimmune diseases, including those affecting the CNS, such as multiple sclerosis (MS) (10–15) or neuromyelitis optica (NMO) (16, 17). **Figure 1** summarizes some of the proposed mechanisms for the reciprocal interactions that occur between the intestinal microbiota and the brain. In this review, we discuss some of the most recent findings that suggest the multifactorial nature of the gut/brain axis in the context of neurological diseases; a topic that has been reviewed in recent years by other authors [for a recent review: (18)]. The manuscript also highlights some key components of the interface between the gut microbiota and the CNS of multiple sclerosis (MS) patients (19). Our review extends the discussions by providing some potential novel avenues for treating neuropsychiatric diseases based on the conditions’ neuroinflammatory components and the influential role of the gut microbiota regulating inflammatory processes.

## Gut Microbes and Microbial Products as Therapeutics Against Neuroinflammation

The intestinal epithelium is a single layer of different cell types that promote communication between the host and the intestinal lumen. Enterocytes serve as absorptive cells that line the epithelium that play a critical role in digestion through the uptake of water, ions, and nutrients. Goblet cells are known for producing mucin proteins that provide a protective layer from the lumen and facilitates the exchange of molecules between the epithelium and luminal environment (20). At the base of the crypts of Lieberkün, Paneth cells secrete antimicrobial peptides and proteins *via* the release of granules (21). In addition, enteroendocrine cells produce neuroendocrine molecules (i.e., gut hormones) that modulate physiological processes within and outside the gut (22). Within the Peyer’s patches, microfold cells (M-cells) surveil the intestinal lumen sampling microbes and microbial components and aid in transporting bacteria to gut-associated lymphoid tissues (GALT) (23). The GALT is the reservoir for almost two-thirds of the total lymphocyte populations within humans and other mammals (24, 25). Because the gut lumen harbors as many as 1 x 10^13^ bacteria as well as a wide range of possible pathogenic species, the presence of vigorous and effective immune cells is imperative for our well-being. As a result, the gut microbiota is an essential regulator of the immune system’s function and the nervous system and may be critical in the control of neuroinflammation (26). The distal small intestine is a reservoir for immune cells expressing and secreting the proinflammatory cytokine interleukin 17 (IL-17). T helper (Th) 17 (Th17) cells are essential in controlling extracellular pathogens and orchestrating neutrophil infiltration; however, they also promote tissue inflammation mediated by IL-23 (27, 28). Intestinal bacteria have been identified as an essential trigger for the differentiation, upon activation, of naïve Th cells into Th17 cells by activating the Retinoic-acid-receptor-related orphan nuclear receptor gamma (ROR-γt) master regulator and the production of proinflammatory cytokines of the IL-17 cytokine superfamily such, IL-22, or granulocyte-macrophage colony-stimulating factor (GM-CSF), among others. Th17 are a necessary adaptive immune cell population against extracellular pathogens by their ability to recruit neutrophils to infection sites. Significant roles for Th17 cells have been described for several autoimmune conditions, including human MS (29), in response to the microbiota (30). Th17 are critical in the induction of many experimental models of human diseases such as experimental autoimmune encephalomyelitis (EAE), the experimental model of multiple sclerosis and other disease models such as rheumatoid arthritis and experimental uveitis (31).

Among the microbes capable of initiating a Th17 profile, the most widely studied is the *Segmented filamentous bacterium* (SFB). SFB act as commensal symbionts by inducing Th17 cells in the small intestines (32, 33) and other immune compartments where autoimmune inflammation occurs (34, 35). During “leaky gut”, when the intestinal barrier is compromised, microbial products disseminate systemically, leading to activation of the IL-23 inflammatory pathway promoting Th17 responses and barrier repair (36). Leaky gut may be caused in part by specific metabolic, toxic product (fragylysin) that is produced by gut commensal bacteria, in particular, *Bacteroides fragilis* (37, 38). Fragylysin produced by enterotoxigenic strains *B. fragilis* is a zinc metalloproteinase with effects in the intestinal barrier integrity as it functions as an endotoxin, promoting sepsis in mice (37). The cleavage of E-cadherin by fragylysin was shown to be the underlying mechanism triggering leaky gut but pathogenic *B. fragilis*. As the lipid A present in LPS produced by other Gram-negative bacteria, fragylysin promotes strong pro-inflammatory responses that can result in neuroinflammatory processes and associated with CNS diseases, such as Alzheimer’s disease (39–41).

Alternatively, the gut microbiome is involved in the critical role of immune homeostatic balance, *via* the promotion of Th cells that induce peripheral T regulatory (iTregs) expressing the transcription factor forkhead box P3 (Foxp3). Tregs produce various antiinflammatory cytokines including IL-10 and Transforming growth factor-beta (TGF-β), and are recognized for their ability to control inflammatory cell subsets’ proliferation. Tregs appear to be critical in both the protective response in experimental EAE and dysfunctional in those with MS patients with reduced suppressive potency (42) and frequencies in the peripheral blood obtained from MS patients, as well as in Alzheimer’s disease patients (43). Several bacterial taxa promote the expansion of iTreg populations. Polysaccharide A (PSA)-producing *B. fragilis* (44), Lactobacilli (45), specific clusters of Clostridia (46), or *Prevotella histicola* (47), among others, expand iTregs and have been associated with neuroprotection in experimental models of disease (47–50).

One key mechanism of protection observed in different CNS disease models is the production of interleukin 10 (IL-10). IL-10 is an antiinflammatory cytokine member of the class II superfamily found as a homodimer produced by monocytes, NK cells, B cells, and different subsets of activated T cells, including Tregs. Tregs induced in response to intestinal microbes may contribute significantly to the control of CNS inflammatory diseases. Experimentally, much of the work done to elucidate the microbiota’s impact on IL-10-mediated control of CNS inflammation is in experimental autoimmune encephalomyelitis (EAE) in mice or rats. EAE is induced either passively by the adoptive transfer of autoreactive T cells or actively by subcutaneous injection of self-antigens. The induction of disease disrupts peripheral tolerance, which results in the proliferation of self-antigen-specific T cells that differentiate into effector, pathogenic Th1 and Th17 cells within the secondary lymphoid tissues. From there, Th1 and Th17 cells circulate through lymphatics to the bloodstream. T cell activation increases the expression of surface integrins that allow immune driven cells to cross the blood-brain barrier into the CNS parenchyma. In the parenchyma, autoreactive, pathogenic T cells are reactivated by resident antigen-presenting cells, activation that results in exacerbated production of proinflammatory cytokines. Other cells, such as monocytes, neutrophils, dendritic cells, and resident glial cells, contribute to the pathogenesis and progression of the disease by mechanisms yet to be elucidated entirely (51). Germ-free mice exhibit reduced disease severity that is reversed upon colonization with SFB (34).

In CNS inflammatory processes, such as those observed in patients suffering from multiple sclerosis (MS), the suppressive capacity of IL-10 has been associated with dysbiosis (14, 15). Experimentally, IL-10 elicited by regulatory cells upon exposure to intestinal microbes such as *B. fragilis* PSA promotes protective responses against neuroinflammation (49, 50, 52). The induction of IL-10 by PSA has been also shown in human *in vitro* systems in mechanisms dependent on antigen presentation by dendritic cells and induction of Tregs (53), in CNS autoimmunity (54). More recently, the protective effects of IL-10 induced in response to the oral treatment with PSA were confirmed against viral encephalitis (55). In their work, Ramakrishna and colleagues show that the polysaccharide is protective against Herpes Virus Encephalitis (HVE) mediated neuroinflammation induced in mice through IL-10 produced by CD4^+^ T cells with a regulatory phenotype (ICOS^+^CD39^+^CD37^+^), CD37^+^CD8^+^ T cells, and IL-10-producing B cells. PSA’s protective effects were observed after early treatment against neuroinflammation and were also observed in HVE mice treated orally with PSA-producing *B. fragilis* (55). An accumulation of IL-10-producing regulatory cells was observed in the cervical lymph nodes of treated mice when compared with PBS-treated sham controls (55). Rojas et al. found that commensal-reactive immunoglobin A (IgA)-producing plasmablasts and/or plasma cells are reduced in EAE mice’s gut and act as an essential source of IL-10, suggesting that IL-10 expression is required to attenuate EAE symptoms (56). Further, the study demonstrates that IgA-producing B cells in the gut can enter the periphery and then infiltrate inflamed CNS tissues, providing evidence for the reduction in IgA targeted fecal bacteria during MS relapses (56).

The experimental evidence gathered in recent years using various animal models of CNS disease highlight the importance of regulating the immune responses to gut microbes. Understanding the balance of effector Th cells, such as Th17, Th1 cells and Tregs, and regulatory B cells, during homeostatic and immune responses in the intestinal microbiome may shed light on psychiatric disorders.

## Clinical Evidence That Suggest the Value of Exploring the Microbiome in the Context of Psychiatric Disorders

The apparent rise in mental health concerns constitutes a broad socioeconomic and clinical problem in developed and underdeveloped countries (57). The impact of neurological disorders affecting mood and behavior impacts interpersonal relationships. Although these diseases’ etiological nature is mostly unknown, it is appreciated the CNS of those patients suffering from a psychiatric disease have both neurological alterations and an inflammatory component that may trigger or exacerbate these conditions (58).

Among the broad list of symptoms that characterize psychiatric disorders, gastrointestinal tract dysfunction is not uncommon. The enteric nervous system (ENS), as a fundamental part of the autonomic nervous system, plays a crucial role in human physiology by regulating the GI’s autonomous actions. Although the autonomous nervous system innervates it, the ENS can function independently using afferent and efferent neurons and interneurons, forming what is known as the second brain. The ENS is critical to regulating peristalsis, enzyme production, and the synthesis of neurotransmitters such as serotonin, dopamine, or acetylcholine, among many others (59). In turn, these physical and chemical factors serve as modulators for microbial growth: for instance, peristaltic movements regulate nutrient flow through the GI tract, influence the nutrient and oxygen availability for gut microbes.

Notably, the ENS can directly communicate with the CNS through the parasympathetic and sympathetic nervous systems. Recent studies show that enteroendocrine cells of the gut epithelium secrete hormones and communicate with the brain through electrochemical signals. By having axon-like basal formations that contain neurofilaments (60, 61), enteroendocrine cells through synapses and the secretion of glutamate, connect with vagal nerve cells and stimulate nutrients to the brain (62). More recent work demonstrates that the system is associated with dopamine activity and reward circuits in rats linking the gut vagal nerves with the striatum in the brain (63). The neuroendocrine system is another mechanism for reciprocal control of the gut-brain axis, including the hypothalamic-pituitary-adrenal (HPA) axis. Through the HPA axis, environmental factors such as stress regulate intestinal physiology, the immune system, and metabolic system (64). Human cortisol produced in response to the HPA axis activation is a major modulator of the intestinal microbiota and a regulator of intestinal permeability (65). In turn, inflammation and imbalanced levels of neurotransmitters have been proposed as inhibitors of the negative regulation that controls the release of cortisol (66).

Co-morbidity of functional gastrointestinal disorders is more frequent in patients that suffer from psychiatric disorders than those considered mentally healthy (67). Inflammatory bowel syndrome (IBS) and inflammatory bowel diseases (IBD) have been linked to mental health disorders over the last decades, as evidenced in a review discussing ten published case-control studies conducted by Shah and colleagues indicating a higher prevalence of IBS and ulcerative colitis (UC) in patients suffering from anxiety or depression than in control individuals (68). Moreover, the study showed that the association between the mental disorder and the GI dysfunction appears to be reciprocal since the severity of the mood diseases’ symptoms increases in IBS and UC patients (31). The review highlights a fundamental question that remains to be answered. Are GI diseases cause or consequence of the bidirectional interaction between the brain and the gut?

Another symptom associated with mental health disorders that suggest a reciprocal interaction between the brain and the gut is functional constipation (69, 70). The reduction in the flow of fecal content within the gut impacts significantly the environmental conditions that directly affect microbial growth and could affect the composition of the gut microbiota. As a result, the CNS pathological process would determine the proportions of aerobes versus anaerobes or the relative abundance of microbes based on nutritional requirements within the large intestine that may be considered a vital constipation factor. The interactions with the gut epithelium and underlying immune system could then affect inflammatory processes, including those within the CNS. Furthermore, the microbiota composition changes could modify the composition of metabolites, including short-chain fatty acids (SCFAs) and neurotransmitter consumption and production, and others. The link between constipation and psychiatric diseases could be another instance of the bidirectional association between the CNS and the gut, as previously hypothesized by us and others (71). Using the EAE model of inflammatory demyelination, our group demonstrated the reciprocal interactions in the context of the gut/brain axis and disease (72). While EAE induction altered the composition of the gut microbiota at the early stages of the disease, alterations that correlated with disease severity levels, changes in the microbiota with a high dose of a mixture of a broad spectrum of antibiotics resulted in reductions in the severity of EAE (72). Others had previously shown that the disease induction increased intestinal permeability and proinflammatory responses in the gut (73). Increased intestinal permeability, often referred to as leaky gut, can be reversed with the probiotic treatments (74), and in the context of disease through fecal transplantation (75). Although the mechanisms linking disease and intestinal permeability remain elusive, it has been demonstrated that gut dysbiosis promotes leaky gut (76). In turn, a commensal microbe such as *B. fragilis* can secrete neurotoxic (fragilysin) that can lead to a biologically “leaky gut” (38). An increased serum IgG and IgA levels produced in response to LPS were observed in the context of depression, when 112 diagnosed patients were compared with 28 healthy individuals suggest the translocation of microbial-derived metabolites from the intestinal lumen to circulation, enhanced in the context of mental diseases (77). These findings suggest, once again, the multifactorial nature of the changes that occur in the intestinal ecosystem in health, but more intriguingly, during CNS disease.

Although the experimental findings suggest an essential association between GI dysfunction and brain diseases, the clinical evidence has not yet been solidified. One significant pitfall that case-control studies face when comparing the microbiota of patients of psychiatric disorders with healthy controls is precisely determining a healthy individual for comparison. Considering that inflammation and neuroinflammation might be considered an essential modulating or even triggering factor for mental health diseases, identifying mentally healthy individuals constitutes a significant challenge due to our constant exposure to inflammatory environmental and internal factors that could potentially promote changes in the microbiota. Additionally, the permanent exposure to confounding factors such as pollution, stress, diet, and many others, result in major difficulties grouping individuals when inclusion and exclusion parameters are considered, such as age, sex, pharmacologic therapies or use of antibiotics in the studies. Nevertheless, despite limitations such as sample sizes, the clinical data gathered to date suggests a link between mental health diseases and microbiota. A large study involving 871 mothers and children performed by interview and questionnaire evaluated the impact of using antibiotics in children during the first year of age and from year one to three and a half, and their neurocognitive outcomes (78). Approximately 70% of the children used antibiotics during the time-period studied. Intelligence scores obtained at ages 3 ½, 7, and 11 indicated an increased rate of behavioral difficulties and depression-related symptoms in those receiving antibiotic treatment (78). The results obtained in this study imply that early disruption of the intestinal microbiota could result in neurological disorders associated with psychiatric conditions. This bidirectional effect of dysbiosis on the gut-brain axis has been assessed in several affective and psychiatric disorders, as discussed below.

### Autism Spectrum Disorder

Autism affects mental development that impacts communication and behavioral patterns, which may appear first in children aged two or younger. As noted in the Diagnostic and Statistical Manual of Mental Disorders and derived works, the patient has “difficulty with communication and interaction with other people, restricted interests and repetitive behaviors, and symptoms that hurt the person’s ability to function properly in school, work, and other areas of life” (79). Although genetics is a risk factor, the causes of the disorder remain unknown. Evidence suggests that germ-free mice have social and repetitive behavior deficits, indicating that the gut microbiota composition is required for social development (80, 81). Environmental triggers, including changes in the intestinal microbiota, are currently the focus of investigation (82). As discussed previously with mental disorders, ASD has a known association with GI dysfunction (83).

Recent studies in experimental ASD support a link with the gut microbiota (84), and microbial products of intestinal fermentation such as SCFA (85, 86), maternal diet (87), and immune dysfunction (88). Colonization with *B. fragilis* discussed earlier, corrects symptoms of maternal immune activation (MIA) used as a model for ADS by restoring intestinal integrity and re-balancing the composition of the dysbiotic following MIA induction (84). The gut/brain link in ASD is also strongly supported by findings using a genetic model of the disease that suggest the importance of the microbiota regulating the disease, as results showed that in the diseased animals, a reduction in *Bifidobacterium* and *Blautia* species impacted tryptophan and bile-acids metabolism that in turn resulted in increased intestinal permeability and exacerbated ASD behavioral symptoms (89). Moreover, the treatment of ASD mice with *Lactobacillus reuteri* reversed social deficits in a mechanism controlled by the vagus nerve’s stimulation and depended on the expression of oxytocin receptors (90).

While experimental models of the disorders suggest the existence of the link between the microbiota and disease, clinically mixed results support the possible crosstalk between the intestinal microbiota and ASD. Some of those studies reported different taxonomical profiles in the microbiota of ASD patients compared with non-ASD children (75, 76, 79, 82–84), while others did not support the association when comparing the microbiota of ASD children with siblings sharing environment (91, 92). The presence of *Clostridium perfringens* was also used to link ASD with the microbiota. In children with ASD, 49 isolates of *C. perfringens* were detected (29 children), versus 30 that were isolated from 17 healthy individuals and 32 detected in 24 obese children (93). Moreover, the *cpb2* gene expression that encodes *C. perfringens*’ Beta2 toxin was significantly enhanced in ASD children (93). Despite the discrepancies, reduced alpha diversity of the microbiota and reduced beta abundance of specific bacterial species have been reported, and as it will be described later, promising results have been obtained in one fecal microbiota transplantation (FMT) pilot project (94). The experimental evidence may also support the use of probiotics or prebiotics. An intervention based on the use of prebiotics and exclusion diet was recently tested in 30 ASD children, resulting in expected microbiota changes, but more importantly, reduced abdominal pain and reduced bowel movement (95). A probiotic intervention reduced plasma myeloperoxidase (MPO) levels, an inflammatory mediator produced by polymorphonuclear leukocytes during oxidative burst, in ASD children (96). These findings could provide further evidence for the link between the control of intestinal inflammation and disease in ASD (96).

### Schizophrenia Spectrum Disorders

Schizophrenia is a mental health disorder that impacts the individual’s ability to interact with the environment and others. The disease is considered chronic and severe, with symptoms that may arise during puberty or later until about age 30. It appears that an imbalance in the brain’s neurotransmitter profiles is present in schizophrenic patients (97). Although family history is a risk for the disease, environmental factors, including viral and parasitic infections, are under scrutiny (98). Neuroinflammation has also been recently proposed to play a role in the pathology of schizophrenic brains (99, 100), including the impact of Th17 cells in 22q11.2 deletion syndrome (22q11.2DS) patients suffering from schizophrenia spectrum disorders (SSD) and psychotic symptoms (101). A positive correlation between IgG levels to dietary wheat gluten and bovine milk casein in serum and cerebrospinal fluid of schizophrenic patients was found (102). In patients suffering from early-stage schizophrenia, the combination of risperidone with the antibiotic minocycline resulted in improved outcomes in Assessment of Negative Symptoms (SANS) and Positive and Negative Syndrome Scale (PANSS), indicating the potential of supplementing the treatments with antibiotics as a new therapeutic avenue for schizophrenia (103).

The importance of the intestinal microbiota regulating immune homeostasis could then be a relevant factor to consider in schizophrenia (104). A recent study examined the composition of the fecal microbiota of 28 first-episode psychosis (FEP) patients, 14 of which were clinically diagnosed with schizophrenia. Results suggested that bacterial numbers, specifically Lactobacilli, *Lachnospiraceae*, *Ruminococcaceae*, and *Bacteroides* spp. were correlated with symptom severity in patients, with predominant bacteria strongly correlated to negative symptoms and poorer function. Moreover, *Lactobacillaceae*, *Halothiobacillaceae*, *Brucellaceae*, and *Micrococcineae* were increased, and *Veillonellaceae* were decreased in FEP patients compared to controls (105). Because FEP patients are vulnerable to relapse, understanding intestinal microbiota’s role in immune function may be useful for the development of precluding strategies and novel treatment targets. When comparing serum cytokine profiles of schizophrenic patients to healthy controls, schizophrenic patients displayed elevated serum levels of inflammation, as seen with an overall decrease in the anti-inflammatory IL-2 and an increase in IL-6, IL-8, and TNF-α (106).

Using a rodent model of schizophrenic-like behavior known as subchronic phenylcycline (subPCP) treatment, Jørgensen and colleagues investigated the possible effect of subPCP on the gut microbiota and found slightly significant alterations in the core microbiome of subPCP and vehicle-treated rats. Additionally, differences in microbiota profiles were associated with poor object recognition memory performance, suggesting gut dysbiosis impacts cognition (107). Furthermore, studies suggest that patients with schizophrenia have increased soluble CD14 (102) and increased intestinal permeability (108), demonstrating evidence of bacterial translocation and intestinal inflammation. Another study introducing a probiotic treatment inhibited pro-inflammatory pathways and ameliorated gut dysbiosis caused by yeast (109). These data provide profound evidence that alterations to the gut microbiome may impact immune responses and be an alternative avenue to explore future therapeutic interventions. Mechanistically, a recent study provides further evidence for the hypothesized gut/brain connection in the context of schizophrenia. In mice, the transplantation of fecal content from schizophrenia patients to a murine model of the disease resulted in exacerbated symptoms associated with the disease, such as psychomotor hyperactivity and learning and memory dysfunction (110). Remarkably, the authors of the study highlighted the importance of tryptophan-kynurenine metabolic pathways associated with disease-driving microbiota.

### Post-Traumatic Stress Disorder

PTSD is a mental health disease caused by trauma and severe stress, by exposure or witnessing, characterized by symptoms that severely affect negatively social behavior and social interactions. In a study using samples from a South African cohort, although alpha- and beta-diversity of the intestinal microbiota of trauma-exposed control individuals and PTSD patients were not significantly different three phyla were found to be reduced in PTSD stool samples: *Actinobacteria*, *Lentisphaerae*, and *Verrucomicrobia* (111).

### Depression

Depression is a debilitating psychiatric illness that affects individuals of any age and significantly impacts the brain, the immune system, and the endocrine system (112). Clinical depression, also known as major depressive disorder (MDD), causes immense feelings of sadness and hopelessness, anxiety, lack of energy, cognitive impairment, and other symptoms. Monoamine neurotransmitters such as serotonin, dopamine, and noradrenaline play a role in mood changes, anxiety, and depression (113). Antidepressants are at the forefront of treatment by increasing levels of these neurotransmitters to reduce depressive symptoms. Because there are limitations to the response on antidepressants (114), other factors may play a role in the disease. Traditionally, depression studies have focused on behavioral, genetic, and neurological pieces of the disease. More recently, environmental factors, synaptic dysfunction, hyperactive HPA axis, neuroinflammation, and gut dysbiosis have been explored. Elevated serum levels of IL-6, a potent activator of HPA and a pleiotropic inflammatory cytokine, in MDD patients supports that immune homeostasis is involved (115).

Recent findings support that the composition of the gut microbiota of patients with depression is different from that of healthy individuals. A preclinical and clinical study showed that with an orally administered probiotic formulation (*Lactobacillus helveticus* R0052 and *Bifidobacterium longum* R0175), psychological distress was mitigated, suggesting that the gut microbiota play a role in depression, anxiety, and stress (116). The microbiota of patients with depression displays a low-diversity dysbiosis with elevated levels of Bacteroidetes and Proteobacteria, and lower levels of Firmicutes (29).

### Anxiety and Stress

As previously introduced, the HPA axis is a significant regulator of the mammalian physiology. Through the HPA axis, the neuroendocrine system modifies the secretion levels of cortisol (in humans, corticosterone in rodents) in response to stress levels. Stress triggers the secretion of corticotropin-releasing factor (CRF) in the hypothalamus. CRF stimulation of the anterior pituitary gland promotes adrenocorticotropic hormone (ACTH) secretion that stimulates the release of cortisol in the adrenal cortex. The release of cortisol increases with stress for several hours until reaching a serum concentration that triggers a negative feedback response shutting down CRF release in the hypothalamus. Cortisol release depends on factors such as sex and age, but other confounding factors have been identified, such as inflammation, obesity, drugs, and other factors (117), also known to affect the composition of the microbiota. Some of those factors, such as inflammation and neurotransmitter balances are also induced by alterations of the microbiota.

## Discussion and Novel Therapeutic Directions

The experimental and clinical data summarized above provide increasing evidence for a multifactorial connection between the intestinal microbiota and the CNS in health and disease. However, uncertainties that remain to be addressed. Whether a disease is preceded by changes in the microbiota or alternatively dysbiosis results from ongoing CNS disease is an essential question that requires further exploration to be answered. Furthermore, whether the microbiota is altered differently at different disease stages, periods of relapses and remissions need to be addressed. Exploring this question could provide insights into whether the observed microbial changes’ relevance as a biomarker approach to predict clinical outcomes or even responsiveness and unresponsiveness to therapies affecting the microbiome.

The search for most appropriate microbiome-based therapeutic approaches and whether the approach is disease-specific needs further investigation. Targeting the microbiota for the treatment of CNS inflammation has been previously used as minocycline (118). Treatment with minocycline reduced T cell activation, proliferation, and production of inflammatory cytokines (119, 120), reduced microglial activation (121), and reduced axonal loss and neuroprotection in animal models of CNS inflammatory demyelination (122). Interestingly, minocycline inhibits the production of matrix metalloproteinases, mediators of intestinal disruption, or “leaky gut” (123). In EAE, the treatment with minocycline is protective (123–125), and several trials were done to determine the efficacy of the treatment against MS [for a review, (118)]. Different therapeutic approaches are currently being explored experimentally (126); This includes treatment with probiotics, purified microbial compounds, fecal microbiota transplantations (FMT), phage therapies, dietary changes, and dietary habits are being evaluated.

### Prebiotics and Probiotics

As noted previously, Eli Metchnikov in the late 1800s suggested that yogurt would be an essential aid in the control of human anxiety. The potential of probiotics was proposed over a century ago in two different clinical studies published in 1909 (127) and 1910 (128). Both studies evaluated the effects of the treatment of melancholia, or depression, with orally administered lactic acid bacteria. Prebiotics, nutritional supplements that favor the growth of proposed beneficial microbes, is now postulated as a novel mechanism to treat behavioral diseases (129). Although probiotics for the treatment of psychiatric disorders have been proposed and discussed (126, 130–134), few studies are sufficiently powered to assess their benefit in humans fully (126). As highlighted in the previous section, a probiotic mixture administered for 30 days to healthy volunteers in a double-blind group comparison with placebo study showed reduced psychological distress quantified by significant reductions in global severity index, somatization, depression, anger-hostility, as well as anxiety-related markers (116). A subsequent addendum to the previous study reported that the administration of the probiotic formulation of *Lactobacillus helveticus* R0052 and *Bifidobacterium longum* R0175 to 25 subjects with low urinary free cortisol (UFC) levels (less than 50 ng/ml at baseline) and on psychological distress resulted in similar improved parameters as those identified previously (135). *Bifidobacterium longum* 1714 has also been tested in healthy volunteers and reported beneficial effects against depression and increased memory (136). A recent randomized trial performed in 75 children that received either *Lactobacillus rhamnosus* GG or placebo showed promising evidence that early supplementation with probiotics (during the first 6 months of life) could reduce later development of attention deficit hyperactivity disorder and autism spectrum disorder diagnosed at age 13 (137). Although encouraging, the sample size is limited and larger groups are needed to be conclusive, as indicated by the authors of the study (137). Other studies reported beneficial effects of different probiotic formulations against stress (138, 139), while others failed to provide positive outcomes of mental health in healthy individuals (126, 140).

### Purified Microbial Components and Metabolites

One observation made clear in light of all recent efforts to characterize the intestinal microbiota taxonomically and functionally is its complexity. The intraindividual and interindividual variability of the microbiota is high and changes over time due to, therapeutic administration, infection, and exposure to many other environmental factors. Moreover, some microbial species within our intestines form intrinsic functional networks that, when disassembled, results in functional loss. Because of that, a general therapeutic approach that does not consider this diversity in taxa and function might not be sufficient to promote protective effects against a given condition or stage of the disease. A possible alternative would be selecting a known microbial compound, expressed or produced and secreted, capable of inducing a response with a defined mechanism of action against neuroinflammation and neurodegeneration. It this scenario, the intestinal microbiota is considered a new source for novel therapeutics (141). For example, PSA produced by *B. fragilis* and purified in the laboratory has shown protective effects after oral treatment against neuroinflammation in different experimental disease models by inducing antiinflammatory IL-10-producing regulatory T and B cells (49, 55, 142). The zwitterionic capsular polysaccharide is taken by antigen-presenting cells, dendritic cells (128), and plasmacytoid dendritic cells (143) through, at least partially, recognition by toll-like receptor 2 (TLR2) (44, 142). Dendritic cells then promote T cells’ differentiation with regulatory phenotypes and B cells that produced enhanced levels of IL-10 (44, 49, 55). Although most PSA studies are in experimental models of disease, PSA’s immunomodulatory effects have also been described using human cells (53), and more significantly, cells isolated from patients suffering from neuroinflammation such as in MS (54).

SCFA produced by fermenting bacteria in response to complex carbohydrates has also shown compelling evidence for their potential use against neuroinflammation. It has been shown that dietary habits with increased fiber consumption significantly impact the plasma concentration of proinflammatory cytokines (144). As a result of the microbial metabolism, fermented fibers by the intestinal microbiota result in lactate, formate, butyrate, acetate, butyrate, propionate, among others. Although dietary fibers are the main source for SCFA, mucin produced by intestinal goblet cells is also a source (145). The SCFA are recognized by Free Fatty Acid receptors 2, and 3 (FFA-2/3) expressed in many different cells of different systems, including the immune system, playing a role in various metabolic and immune processes (146). The effects of SCFA have also been observed in the microglia’s maturation, indicating their importance regulating CNS’ function (147). Different mechanisms of action that could lead to protection against neuroinflammation have been described for SCFAs. Butyrate is an essential regulator of the intestinal barrier’s integrity, an anatomical fence that, when disrupted, could impact systemic immune homeostasis (148) and promote disease (149). The reconstitution of germ-free mice with fermenting bacteria that produce SCFA restores the integrity of the blood-brain barrier (BBB) that appears reduced in the absence of microbiota (150). Also, SCFA promotes a regulatory phenotype on T cells by their signaling through G protein-coupled receptors (GPCRs), such as GPR109A, expressed in adipocytes and immune cells. SCFA promotes differentiation of colonic Tregs (151–153) through the inhibitor of histone deacetylases enhancing Foxp3 expression when supplemented with TGF-β stimulation. Butyrate stimulates intestinal epithelial cells to produce TGF-β that, at least in part, could promote Treg differentiation (154). The effects of SCFA in inflammation are thus multifactorial, and although anti-inflammatory functions have been described, more profound evaluation is needed to fully understand their impact on systemic inflammation and neuroinflammation (155).

Other microbial metabolites produced in the intestine might constitute novel targets for therapeutic interventions against neuroinflammation. For example, the levels of tryptophan and serotonin levels are impacted in germ-free animals when compared with conventionally-housed counterparts with significant effects on depressive behavior (156). In this study, the investigators evaluated the depressive disorder model in response to acute tryptophan depletion in the brain, which results in low levels of both tryptophan and serotonin, in the absence or presence of intestinal microbiota. When no microbiota is present, the intervention resulted in an enhanced depressive phenotype, suggesting a link between the microbiota and tryptophan homeostasis in the context of psychiatric disorders (156). Brain-derived neurotrophic factor levels are also reduced in germ-free mice and show increased stress responses than conventional animals, a phenotype that is reversed by monocolonization with *Bifidobacterium infantis* (157). The intestinal microbiota could also be a novel source for therapeutics based on the modulation of neurotransmitter metabolism. For instance, both γ-aminobutyric acid (GABA)-producers and consumers are found in the GI tract. One hypothesis would be that dysbiosis could promote an imbalance on GABA producers and consumers’ levels, thereby promoting an exacerbation of neurological symptoms (158–160). For instance, reduced serum GABA levels have been found in patients suffering from secondary-progressive MS (161), while exogenous GABA administration results in the protection against CNS inflammatory demyelination in EAE mice (162).

### Fecal Microbiota Transplantations and Microbiota Transfer Therapy

One open-label clinical study showed that FMT reduced the symptoms of autism in ASD children between ages 7 and 17 (94). The study showed that in ASD children, the intestinal microbiota diversity was reduced at baseline compared to controls. The study included the pre-FMT treatment of patients with antibiotics for 2 weeks and subsequent doses with microbiota for 10 weeks and a follow-up evaluation 8 weeks after the end of the treatment. The use of vancomycin as a pre-treatment was used to, according to the authors, suppress pathogenic bacteria. The alterations of the intestinal microbiota using antibiotics were previously shown to promote significant improvement in ASD-associated symptoms. The potential benefit of antibiotics has been demonstrated when vancomycin was used to treat a small cohort of ASD children that showed short term improvement in ASD symptoms (163).

In the study of Kang et al., the transplanted microbiota consisted of a standardized human microbiota preparation previously described for the treatment of recurrent *Clostridium* infections (151). At the end of the treatment, the microbial diversity of ASD children treated with the standardized microbiota increased in 16 out of 18 patients during treatment. Interestingly, two patients remained unresponsive to FMT. The study reported an 80% reduction in GI symptoms that included constipation, diarrhea, or abdominal pain, and improved behavioral ASD symptoms in patients receiving the FMT treatment (94). More recently, Kang and colleagues performed a follow-up study 2 years after the Microbiota Transfer Therapy (MTT) with the same 18 participants and concluded that improvements to GI distress were sustained, and autistic-like behaviors were improved (164). The individuals with ASD also exhibited significant increases in bacterial diversity and relative abundances of *Bifidobacteria* and *Prevotella*, providing evidence for MTT as a potential therapy for children with ASD and GI issues (164). The authors acknowledge the study’s limitations in terms of sample size, use of antibiotics and pre-treatment with proton pump inhibitors, and the nature of the comparisons as an open-label analysis, with no placebo control. Other work by Sharon and colleagues found that fecal transplantation from human donors with autism spectrum disorders into germ-free mice promoted hallmark autistic behaviors (165). Nevertheless, the results provided are encouraging and suggest a potential mechanism for the treatment of behavioral disorders.

## Conclusions

Increasing experimental and clinical evidence suggest that the intestinal microbiota and the CNS are connected by a reciprocal gut-brain axis with multifactorial components. Despite the difficulty of understanding the complexity of the interactions between our metabolic, immune, and nervous systems and the microbiota, and between the enormously diverse microbial population of the gut, it is now appreciated the relevance of the interactions for health and disease. Studies in germ-free animals, animals treated with antibiotics, the use of probiotics, monocolonization studies, or fecal transplantation, among other approaches, show that the alteration of the microbiota may result in changes in behavioral outcomes. Many questions remain to be answered regarding the mechanisms of actions by which gut microbes could impact mental health. However, the impact on neuroinflammation appears to be a key route for the regulation of disease. In this context, it is now well established that intestinal microbes, and microbial products, modulate the extend of pro- and anti-inflammatory responses that could exacerbate or reduce the immunopathological parameters associated with the disease. Nevertheless, a more profound analysis is necessary to understand the exact mechanism of control, whether the mechanisms are disease-specific and varies with disease stages, or even if the changes quantified in the composition of the microbiota precede disease are a result of it or co-occur. Despite the limitations of what it is known, the gut/brain axis is a complex but exciting area of investigation that has begun to expose intriguing biological interactions between our body and the microbes that inhabit each of us.

## Authors Contributions

JO-R, CR, and LK contributed equally to the manuscript. All authors contributed to the article and approved the submitted version.

## Funding

Research supported by the National Institute of Neurological Disorders and Stroke (NINDS), of the National Institutes of Health under award number 1R15NS107743.

## Conflict of Interest

JO-R serves as a consultant for Symbiotics Biotherapies. LK is a co-founder of Symbiotix Biotherapies.

The remaining author declares that the research was conducted in the absence of any commercial or financial relationships that could be construed as a potential conflict of interest.
